# WDPCP Modulates Cilia Beating Through the MAPK/ERK Pathway in Chronic Rhinosinusitis With Nasal Polyps

**DOI:** 10.3389/fcell.2020.630340

**Published:** 2021-02-01

**Authors:** Yun Ma, Peng Tian, Hua Zhong, Fan Wu, Qining Zhang, Xiang Liu, Hua Dang, Qiujian Chen, Hua Zou, Yiqing Zheng

**Affiliations:** ^1^Department of Otorhinolaryngology, Sun Yat-sen Memorial Hospital, Sun Yat-sen University, Guangzhou, China; ^2^Department of Otorhinolaryngology, First Affiliated Hospital of Sun Yat-sen University, Sun Yat-sen University, Guangzhou, China

**Keywords:** WDPCP, cilia beating, mitochondria, MAPK/ERK pathway, chronic rhinosinusitis with nasal polyps (CRSwNP)

## Abstract

Cilia loss and dysfunction is one of the typical pathological features of chronic rhinosinusitis with nasal polyps (CRSwNP). Tryptophan-aspartic acid (W-D) repeat containing planar cell polarity effector (WDPCP) has been proven to be an essential element for ciliogenesis in human nasal epithelium, but its role in the beating of cilia remains unclear. In this study, we sought to investigate the role of WDPCP and its underlying mechanism behind the dysfunction in the beating of cilia in nasal polyp tissue. We demonstrated WDPCP expression in the epithelium of nasal polyps. We also investigated the MAPK/ERK pathway in primary human sinonasal epithelial cells to explore the function of WDPCP. The air–liquid interface culture system was used as a model to verify the role of WDPCP and the MAPK/ERK pathway in the beating of cilia. With the dysfunction of cilia beating, we observed a low expression of WDPCP in the epithelium of nasal polyp tissues. Within the *in vitro* study, we found that WDPCP was critical for mitochondrial biogenesis and mitochondrial function in human sinonasal epithelial cells, possibly due to the activation of the MAPK/ERK pathway. The mitochondrial dysfunction caused by U0126 or lacking WDPCP could be partially recovered by dexamethasone. The low expression of WDPCP in nasal epithelium could affect mitochondria via the MAPK/ERK pathway, which may contribute to the dysfunction in the beating of cilia in CRSwNP.

## Introduction

Chronic rhinosinusitis (CRS) is a common disease that affects 5.7–16% of the world population (Hastan et al., 2011; Blackwell et al., 2014; Shi et al., 2015; Kim et al., 2016). CRS is divided into CRS with nasal polyps (CRSwNP) and CRS without nasal polyps (CRSsNP), depending on the presence or absence of nasal polyps (NP). CRS has been identified as an inflammatory disease. Cilia cells play an important role in the nasal epithelium. The beating of cilia occurs in a coordinated manner to generate flow for the clearance of debris and pathogens in mucus. The beating of cilia is affected by a variety of factors such as bacterial pathogens (Ferguson et al., 1988), inflammatory cytokines (Lennard et al., 2000), tobacco smoke (Elliott et al., 2006), temperature (Schipor et al., 2006), and pH value (Sutto et al., 2004). Cilia loss and dysfunction is one of the predominant pathological changes present in CRSwNP (Gudis et al., 2012). However, the mechanism of poor ciliation and cilia dysfunction in CRSwNP remains unclear.

The planar cell polarity (PCP) pathway, which is also the non-canonical Wnt pathway, regulates the convergence and extensive movement of cells during embryogenesis and gastrulation of vertebrates (Wallingford et al., 2000; Jessen et al., 2002). Proteins in the PCP pathway control the morphogenesis and motility of multiciliated epithelial cells (Gray et al., 2009; Sittaramane et al., 2013; Park et al., 2015). WD repeat containing planar cell polarity effector (WDPCP) was critical for ciliogenesis in *Xenopus* and controlled cell polarity in *Drosophila* (Kim et al., 2010; Cui et al., 2013). In our previous study, we have discovered that WDPCP was essential for ciliogenesis in human nasal epithelium, but it was still unclear whether WDPCP regulated cilia motility in the human airway (Ma et al., 2017).

The beating of cilia depends on ATP provided by mitochondria (Seminario-Vidal et al., 2011). Decreased energy levels caused by mitochondrial damage is an important reason for ciliary dysfunction in airway diseases (Cloonan et al., 2016; Liu and Summer, 2019). The mitogen-activated protein kinase (MAPK) and extracellular signal-regulated kinase (ERK) signaling pathway is a protein-serine/threonine kinase cascade that includes dual-specificity mitogen-activated protein kinases kinases 1/2 (MEK1/2) that activates the effector kinases ERK1/2. This pathway participates in a variety of nuclear transcription factors and affects cell proliferation, apoptosis, differentiation, inflammatory response, and other biological processes (Liu et al., 2018). Moreover, mitochondrial membrane potential, mitochondrial oxidative stress, and mitochondrial energy metabolism were also modulated by the MAPK/ERK pathway (Zhang et al., 2019). However, studies regarding cilia disorders related to mitochondrial damage in human nasal epithelium remain lacking.

Therefore, the aim of this study was to investigate whether WDPCP plays a role in the function of cilia in human nasal epithelium and to unveil corresponding molecular mechanisms of mitochondrial damage regulated by the MAPK/ERK pathway.

## Materials and Methods

### Human Tissue Procurement

Our study was approved by the Ethics Committee of the Sun Yat-sen Memorial Hospital of Sun Yat-sen University (2018-212), and written consent was obtained from each participant. Both control subjects and patients with CRSwNP, which were 18–65 years old, were recruited from the Department of Otorhinolaryngology at the Sun Yat-sen Memorial Hospital of Sun Yat-sen University from September 2018 to July 2019. Their clinical characteristics are listed in Table 1. The diagnosis of CRSwNP was based on a European position paper on rhinosinusitis and nasal polyps published in 2012 (Fokkens et al., 2012). Patients with any systemic diseases, acute upper respiratory tract infections, or genetic defects such as primary ciliary dyskinesia were excluded from this study. Control subjects had no evidence of sinonasal mucosal inflammation but have underwent sinonasal surgery for the repair of cerebrospinal fluid rhinorrhea and optic nerve decompression. Mucosa of ethmoid sinuses from control subjects and polyp tissues from patients with CRSwNP were harvested during surgery, and all the NP patients only used nasal glucocorticoids before sampling. The samples were used for various further analyses as follows.

**Table 1:** Patients' characteristics.

	**Control subjects (*n* = 10)**	**CRSwNP (*n* = 15)**	***p***
Gender (M/F)	6/4	6/9	NS
Mean age (Y)	30.8 ± 2.53	45.67 ± 3.63	0.0061
Smoker	3	8	NS
Allergy^†^	0	5	NS
Asthma^‡^	0	3	NS
**Inflammatory cell count from peripheral blood**			
Neutrophilic (× 10^−9^/L)	8.20 ± 0.57	7.06 ± 0.51	NS
Eosinophilic (× 10^−9^/L)	0.19 ± 0.05	0.39 ± 0.07	0.044

*M, male; F, female; Y, years; NS, not significant*.

† *Allergy was diagnosed by specific IgE level test from peripheral blood*.

‡ *Physician-diagnosed asthma with treatment of β_2_-agonist (n = 2) or β_2_-agonist plus glucocorticoid inhaler (n = 1)*.

### Air–Liquid Interface (ALI) Cultures

The complete method for the preparation of primary sinonasal epithelial cultures can be found in our previous study (Ma et al., 2017). Human sinonasal epithelial cells (HSECs) from the control group were transferred to Transwell inserts (Corning, 0.4 μm) to initiate the ALI culture. Fourteen days were required to obtain the differentiated cilia via the PneumaCult™-ALI Medium (Stem cell). The ALI membranes were collected on Day −0, −7, and −14.

### Quantitative RT-PCR

Quantitative real-time PCR (RT-PCR) was performed on HSECs and clinical samples. β-actin was used as a reference for normalization. Nuclear respiratory factor 1 (NRF1), nuclear respiratory factor 2 (NRF2), transcription factor A, mitochondrial (TFAM), and cytochrome c oxidase subunit 4 (COX4) PCR was performed with the Roche LightCycler 480 Real-Time PCR System using SYBR Premix Ex Taq (Takara). The primer sequences can be found in Supplementary Table 1. Relative gene expression was calculated using the 2^−ΔΔ*CT*^ method.

### Histological Staining

Immunohistochemical staining was performed on the paraffin sections. After dehydration, antigen retrieval, quenching of endogenous peroxides, and blocking, sections were incubated overnight at 4°C with polyclonal rabbit anti-WDPCP (HPA, 044144) at 1:50, monoclonal mouse anti-NRF1 at 1:100 (Abcam, ab175932), monoclonal mouse anti-NRF2 at 1:100 (Abcam, ab62352), monoclonal mouse anti-TFAM at 1:50 (Abcam, ab131607), and polyclonal rabbit anti-COX4 at 1:200 (Cell Signaling, 4850S). DAB and hematoxylin were used for staining.

Immunofluorescence staining was performed for ALI membranes and HSECs. In brief, HSECs were incubated overnight at 4°C with monoclonal mouse anti-beta tubulin IV at 1:100 (Abcam, ab11315) and subsequently with Alexa Fluor 488 secondary antibodies (Invitrogen) at 1:200 at room temperature for 1 h, and 40,6-diamidino-2-phenylindole (DAPI) (10 mg/ml, Sigma-Aldrich) for 10 min.

Mitochondrial structure was detected via fluorescent MitoTracker (Invitrogen), which was cultured with HSECs at 1:500 dilution at 37°C for 1 h. Then, the cells were fixed and stained with DAPI for 10 min. HSECs were observed and imaged with a Zeiss LSM 780 confocal microscope.

### Western Blot Analysis

Total protein from nasal mucosa and HSECs was extracted in RIPA lysis buffer. Protein concentrations were determined by bicinchoninic acid assay (BCA). Samples that contained 30 μg of protein were resolved via SDS–PAGE in 10–12% Tris–glycine gels and transferred onto a polyvinylidene fluoride membrane (Millipore) and blocked with 5% bovine serum albumin (BSA). The membranes were incubated overnight with polyclonal rabbit anti-WDPCP (HPA, 044144) at 1:1,000, monoclonal mouse anti-NRF1 (Abcam, ab175932), monoclonal mouse anti-NRF2 (Abcam, ab62352), monoclonal mouse anti-TFAM (Abcam, ab131607), and polyclonal rabbit anti-COX4 (Cell Signaling, 4850S) at 1:1,000, and then incubated with an appropriate secondary antibody (1:10,000) for 1 h. The relative protein levels were quantified by densitometric image analysis of bands using Fiji (National Institutes of Health) and were normalized against β-actin. Antibody specificity test can be found in the Supplementary Figure 1.

### Scanning Electron Microscopy

The membranes that contained HSECs from the Transwell inserts were fixed with 2.5% glutaraldehyde for 4 h at room temperature. Subsequently, the cells were progressively dehydrated in 30, 50, 70, and 90% ethanol for one time per concentration at 15-min intervals, three times in 100% ethanol for 15 min, and three times in 100% tert-butyl alcohol for 15 min. The filters were then glued onto scanning electron microscopic (SEM) stubs and sputter coated to a thickness of 12 nm by gold palladium. The surface of the Transwell membrane was examined at an accelerating voltage of 10 kV using a Quanta-400 scanning electron microscope (FEI).

### *In vitro* Treatment

HSECs were transfected with siRNA targeted to WDPCP using Lipofectamine 3000 (Invitrogen) following the manufacturer's instructions, and the transfection efficiency can be found in the Supplementary Figure 2. HSECs were stimulated by U0126 (10 μM) or dexamethasone (0.01 mg/ml). After 24 h of incubation, the protein was collected for further experiments.

### Mitochondrial DNA Copy Number Analysis

Total DNA was extracted from HSECs using the QIAamp DNA Mini Kit (Qiagen). The mitochondrial DNA (mtDNA) copy number was determined by amplifying genes encoding genomic DNA and mitochondrial DNA. The mtDNA levels were quantified by quantitative real-time polymerase chain reaction on a Roche Light Cycler 96 (Roche) using HV1 primers (forward: 5′-TTGCACGGTACCATAAATACTTGAC-3′, reverse: 5′-GAGTTGCAGTTGATGTGTGATAGTTG-3′). Nuclear gene β-globin primers (forward: 5′-ACACAACTGTGTTCACTAGC-3′, reverse: 5′-CAACTTCATCCACGTTCACC-3′) were used as a nuclear control. Relative quantification of mitochondrial DNA copy number was calculated after using the 2^−ΔΔ*CT*^ method to obtain the expression fold change.

### Mitochondrial Membrane Potential Measurement

The mitochondrial membrane potential was evaluated with JC-1 fluorescent dye (Beyotime, China). JC-1 displayed red or green fluorescence depending on the mitochondrial potential. Normal mitochondrial membrane potential exhibited red fluorescence, whereas damaged mitochondria exhibited green fluorescence. The HSECs were stained with JC-1 in culture media for 20 min at 37°C and were then washed three times with washing buffer. The mitochondrial membrane potential was measured by flow cytometry (BD Biosciences).

### Measurement of ATP Levels

An ATP Assay Kit (Beyotime, China) was used to conduct the ATP assay. Briefly, HSECs were collected in lysis buffer and centrifuged at 12,000 × *g* for 5 min at 4°C. ATP detection reagent was added into 96-wells, and then the standards and samples were added into the wells and the detection solution was mixed. Chemiluminescence was detected by a Synergy H1 Hybrid Multi-Mode Reader (BioTek, USA). The levels of ATP were calculated based on the standard curve and were normalized to the protein content.

### Cilia Beating Frequency

Both ciliated HSECs in ALI culture and clinical samples collected within 30 min were used for cilia beating frequency (CBF) analysis. The room temperature was controlled at 25°C, and the humidity was maintained at 70–80%, which would not significantly affect the CBF (Kempeneers et al., 2019). A high-speed digital video camera (Basler AG) captured images at 100 frames per second and Sisson-Ammons Video Analysis (SAVA) software (National Instruments) was used for video analysis. Each measurement was obtained three times and recorded 15 s each time.

### Statistical Analysis

Data were presented as mean ± standard error of the mean (SEM) and analyzed using GraphPad PRISM 7 (GraphPad Software). One-way ANOVA, Student's *t* test, one sample *t* tests, and Fisher's exact tests were performed for statistical analysis. A value of *p* < 0.05 was considered statistically significant.

## Results

### Expression of WDPCP and the Cilia Beating Function Were Decreased in Nasal Polyps

**Figure 1:**
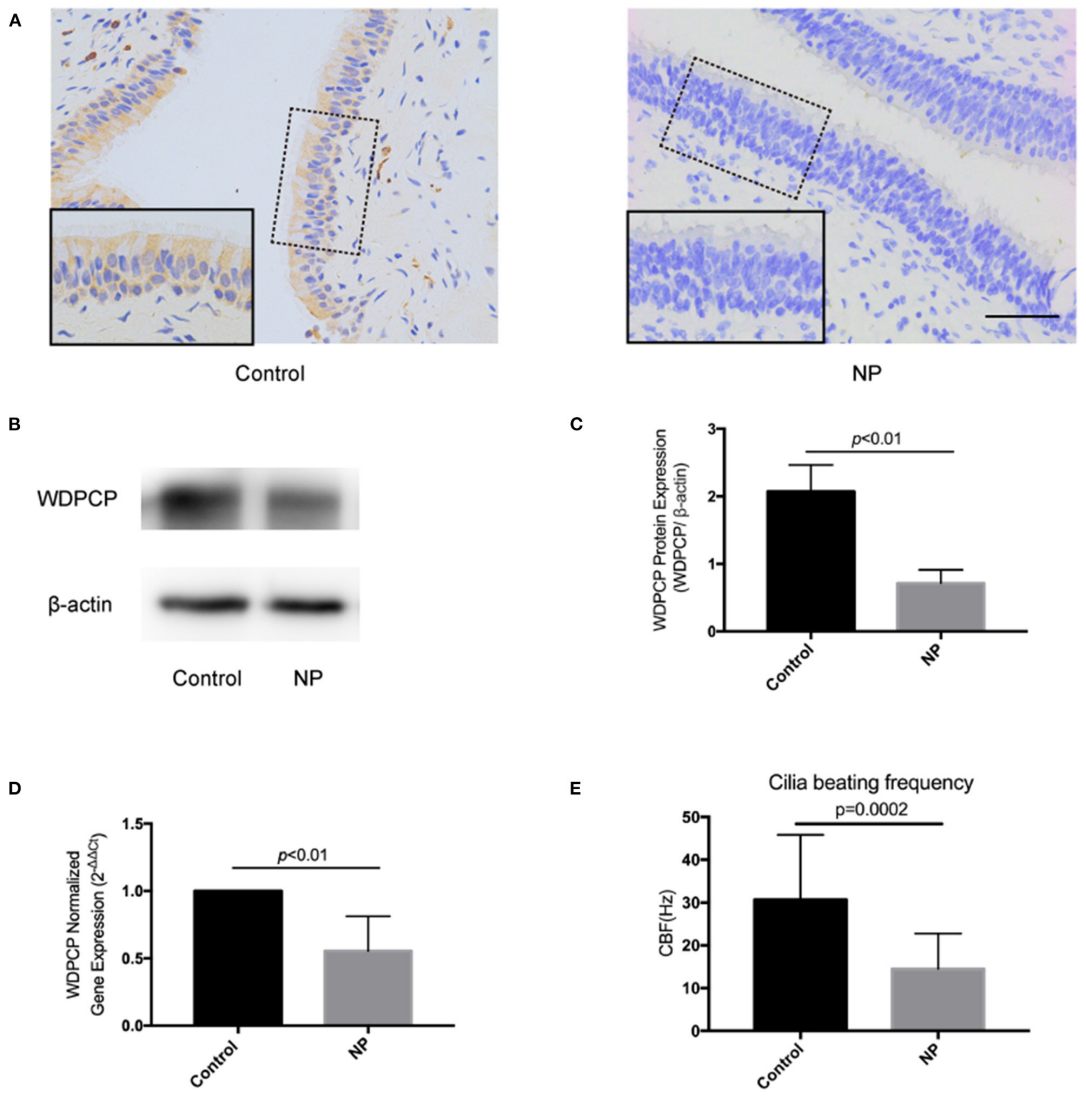
Epithelium of nasal polyps demonstrated a low expression of WDPCP and had decreased cilia beating frequency compared with control subjects. **(A)** The expression of WDPCP in the cytoplasm of epithelial cells from nasal polyps (NP) was decreased as determined by immunohistochemical staining (brown color). Scale bar: 50 μm. **(B,C)** Western blot images and densitometry showed decreased WDPCP protein levels in NP compared with nasal mucosa from control subjects (*n* = 3). **(D)** The copy number of WDPCP mRNA was decreased in NP (*n* = 3). **(E)** Cilia beating frequency (CBF) was significantly reduced in NP compared with control mucosa (*n* = 5).

In our previous study, we have previously discovered that the expression of WDPCP was reduced in the epithelial layer of nasal polyp tissues from patients with CRSwNP compared with control subjects, accompanied with cilia loss (Ma et al., 2017). According to immunohistochemical staining, we discovered that WDPCP was mainly expressed in the cytoplasm of nasal mucosa epithelium and the staining (brown color) was reduced in nasal polyp tissues (Figure 1A). Western blot also showed decreased protein expression of WDPCP in nasal polyp tissue compared with ethmoid sinus tissue from control subjects (Figures 1B,C). Consistent with the protein levels, RT-PCR demonstrated a decrease of WDPCP mRNA levels in nasal polyps (Figure 1D). Moreover, to explore the function of cilia, we measured the CBF and found that the cilia from nasal polys beat at a slower rate compared with control mucosa (Figure 1E). Additional movie files show the cilia beating in more detail (Supplementary Videos 1, 2).

### WDPCP Potentially Acted Through the MAPK/ERK Pathway to Regulate Ciliogenesis and Cilia Function

**Figure 2:**
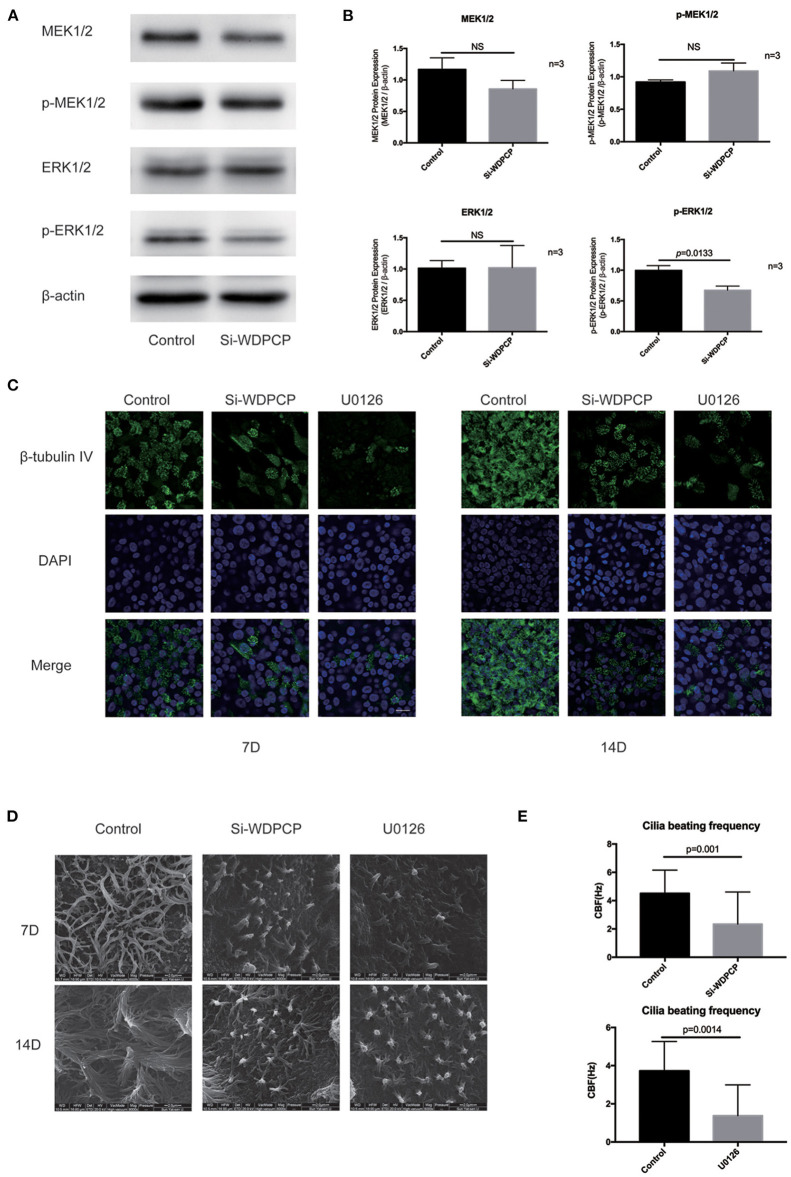
WDPCP regulated ciliogenesis and cilia beating through the MAPK/ERK pathway in HSECs. **(A,B)** The expression of p-ERK1/2 was decreased in Si-WDPCP HSECs (*n* = 3) by Western blot and densitometry analysis. **(C)** Immunofluorescence images showed the decrease of cilia length and quantity in HSECs that lacked WDPCP and U0126-treated HSECs. Scale bar: 20 μm. **(D)** SEM images also showed that the cilia length and quantity were decreased in HSECs that lacked WDPCP and U0126-treated HSECs. Scale bar: 2 μm. **(E)** Cilia beating frequency was reduced on Day-14 in HSECs that lacked WDPCP and U0126-treated HSECs (*n* = 4).

Evidence showed that WDPCP participated in ciliogenesis and could possibly affect the beating of cilia (Kim et al., 2010). To investigate the potential mechanism, we detected the changes of expression in the MAPK/ERK pathway. We silenced the expression of WDPCP in HSECs by transferring small interfering RNAs (Si-WDPCP). Twenty-four hours post-transfection, the expression of phosphorylated ERK1/2 (p-ERK1/2) was significantly decreased in HSECs compared to the control. However, the expression of MEK1/2, p-MEK1/2, and ERK1/2 remained constant after WDPCP silencing (Figures 2A,B).

HSECs could differentiate into respiratory epithelium when they were grown in an ALI. It took approximately 14 days to obtain fully mature cilia. This ALI culture was also used to explore the role of WDPCP and the MAPK/ERK pathway in ciliogenesis and cilia beating. The cilium in HSEC ALI cultures was detected on Day-14 by scanning electron microscopy and immunofluorescence staining. HSECs that lacked WDPCP at the beginning of ciliogenesis differentiated into fewer and shorter cilia compared with the controls (Figures 2C,D). Similarly, HSECs cultured with MAPK/ERK inhibitor U0126 also developed fewer and shorter cilia (Figures 2C,D). We also examined the CBF on Day-14. HSECs treated with Si-WDPCP and U0126 displayed slower cilia beating frequencies compared with controls (Figure 2E). Additional movie files show the cilia beating of HSECs in more detail (Supplementary Videos 3–5).

### WDPCP Regulated Mitochondrial Biogenesis Through the MAPK/ERK Pathway

**Figure 3:**
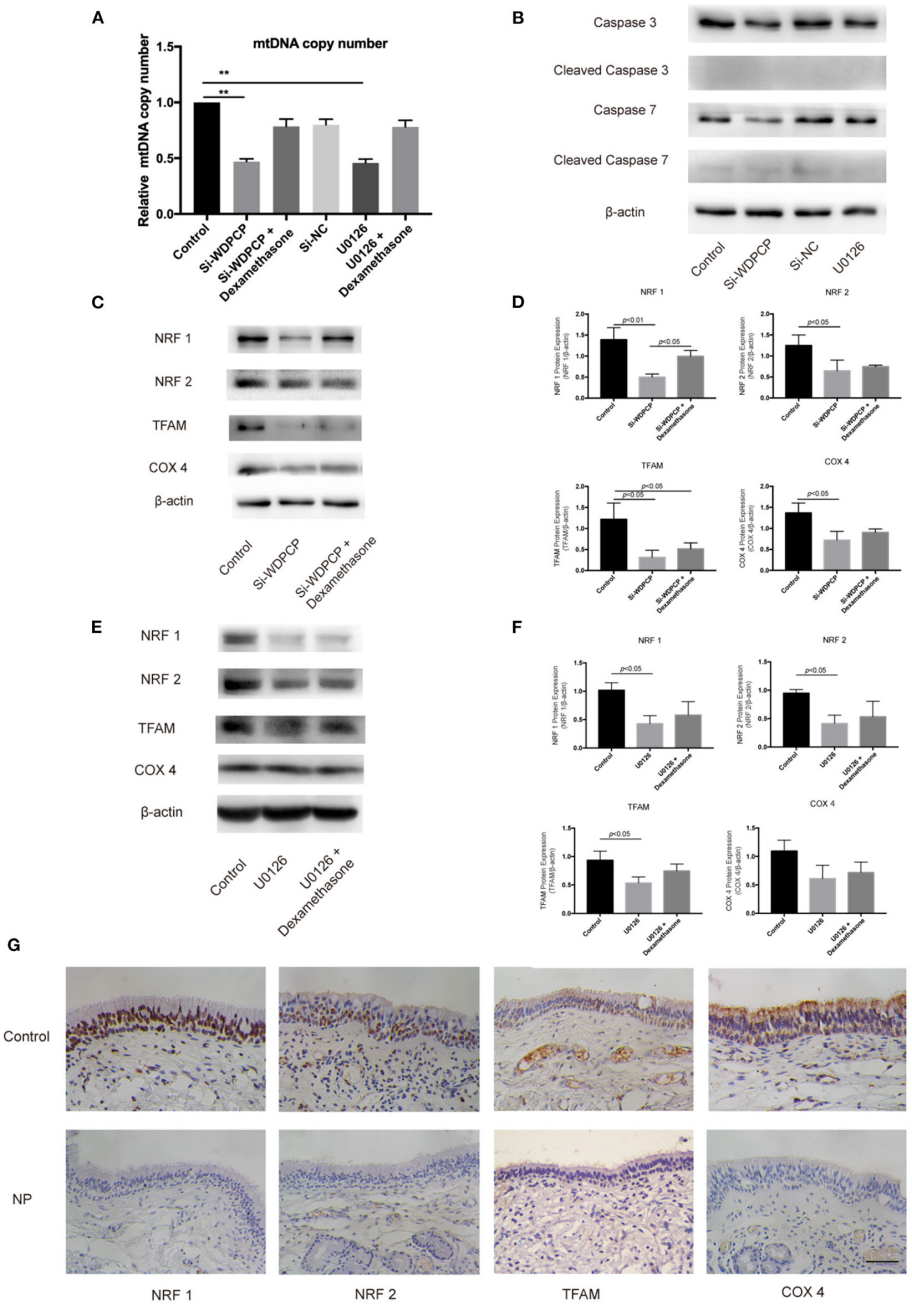
WDPCP silencing or inhibition of the MAPK/ERK pathway could reduce mitochondrial biogenesis. **(A)** mtDNA copy number was decreased in Si-WDPCP HSECs and U0126-treated HSECs (*n* = 3). **(B)** WDPCP silencing or the inhibition of the MAPK/ERK pathway did not activate mitochondrial apoptosis. **(C,D)** NRF1, NRF2, TFAM, and COX4 protein levels were reduced after WDPCP silencing in HSECs by western blot and densitometry analysis. The reduction of NRF1 could be recovered by dexamethasone (*n* = 3). **(E,F)** NRF1, NRF2, and TFAM protein levels were reduced in U0126-treated HSECs (*n* = 3). **(G)** Mitochondrial biogenesis markers NRF1, NRF2, TFAM, and COX4 had low expressions in the epithelium of nasal polyps by immunohistochemical staining. Scale bar: 50 μm.

Substantial evidence suggested that ATP from mitochondria is essential for the beating of cilia (Fliegauf et al., 2007; Workman et al., 2017; Auguste et al., 2018). Thus, we hypothesized that WDPCP acts on mitochondria through the MAPK/ERK pathway to affect cilia beating in CRSwNP. First, we measured the mitochondrial DNA (mtDNA) copy number to observe the number of mitochondria in HSECs. We found that the mtDNA copy numbers were decreased in HSECs after WDPCP silencing or after being cultured with MAPK/ERK pathway inhibitor U0126. Glucocorticoids are known to be the mainstay of medical treatment for CRSwNP. Based on previous research, we explored the effects of glucocorticoids on the mitochondria. mtDNA copy numbers were not significantly decreased after treatment with dexamethasone in Si-WDPCP HSECs and HSECs inhibited by U0126 (Figure 3A).

Decreased mitochondrial biogenesis or increased apoptosis can lead to the reduction in the number of mitochondria. To understand the specific reasons of mitochondrial reduction, we examined the expression of biomarkers for both mitochondrial biogenesis and apoptosis. Western blot results revealed that the apoptosis markers Cleaved Caspase 3 and Cleaved Caspase 7 were not activated in HSECs that lacked WDPCP or in HSECs inhibited by U0126 (Figure 3B). However, we found the low expression of NRF1, NRF2, TFAM, and COX4 in Si-WDPCP HSECs compared with controls (Figures 3C,D). Interestingly, NRF1 protein levels were increased after dexamethasone treatment in Si-WDPCP HSECs (Figures 3C,D). Additionally, the protein expression of NRF1, NRF2, and TFAM could be inhibited by U0126 (Figures 3E,F). Negative control siRNAs (Si-NC) were used to rule out the effect of transfection on mitochondria. Moreover, the decreased expression of NRF1, NRF2, TFAM, and COX4 was also observed in the nasal epithelium and nasal polyps by immunohistochemical staining (Figure 3G).

### The Temporal Mitochondrial Biogenesis in the ALI Culture of HSECs

**Figure 4:**
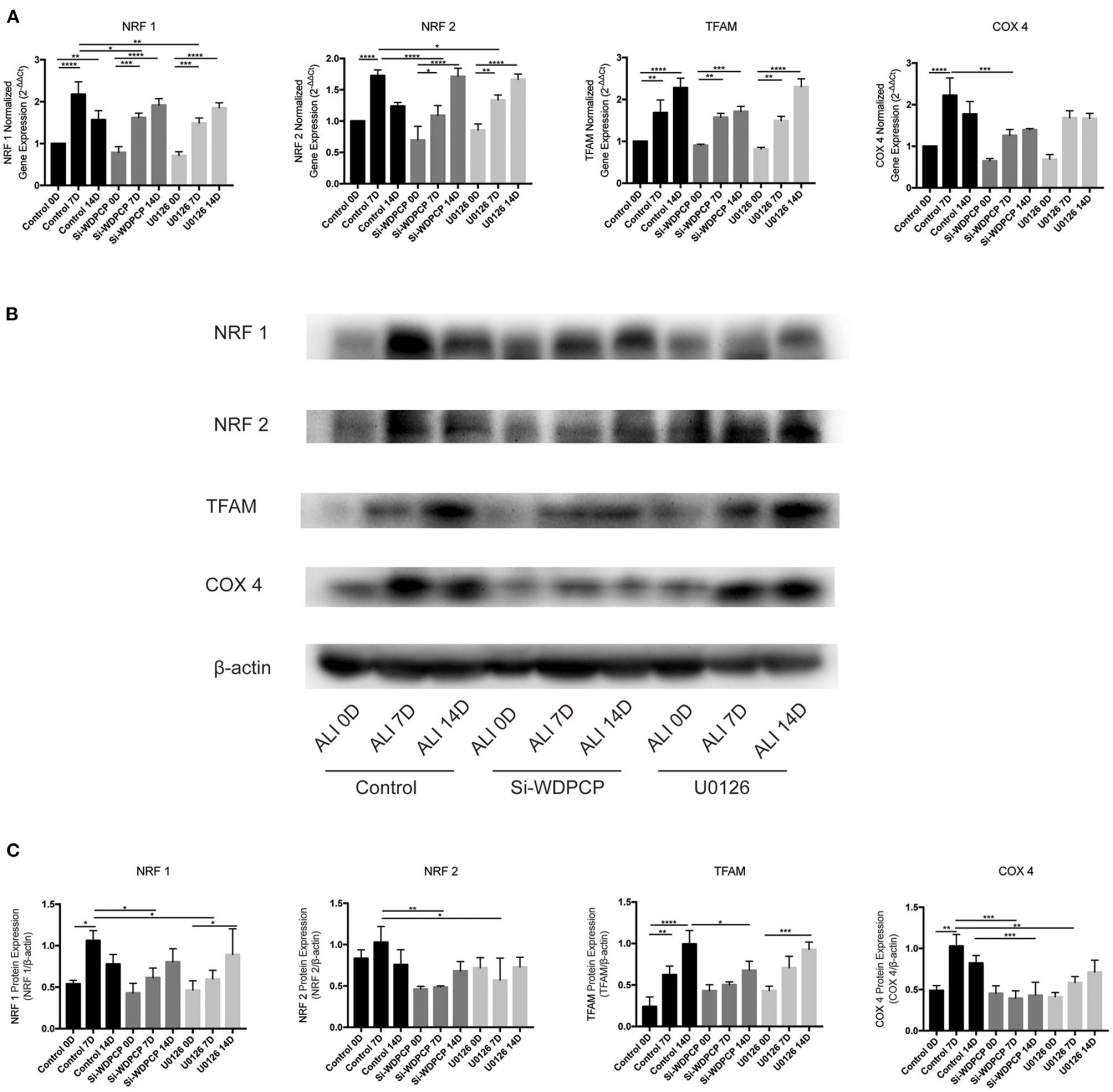
Expression of mitochondrial biogenesis makers in ALI cultures of HSECs. **(A)** NRF1, NRF2, and COX4 mRNA levels increased over 7 days in ALI cultures, but this increase was lesser in Si-WDPCP HSECs and U0126-treated HSECs. TFAM mRNA levels continued to increase over 14 days in ALI cultures (*n* = 3). **(B,C)** NRF1, NRF2, and COX4 protein levels also increased over 7 days in control ALI cultures, and the expression was decreased in Si-WDPCP HSECs and U0126-treated HSECs on Day-7. TFAM protein levels continued to increase over 14 days in ALI cultures, and its protein expression was decreased in Si-WDPCP HSECs on Day-14 (*n* = 3) (*p < 0.05, **p < 0.01, ***p < 0.001, ****p < 0.0001).

The temporal mitochondrial biogenesis in the ALI cultures of HSECs was established to explore the changes in mitochondrial biogenesis during ciliogenesis. Our results showed that NRF1 and NRF2 mRNA and protein expression increased on Day-7 compared with Day-0 and then decreased after 14 days in the control ALI culture. In contrast, Si-WDPCP HSECs and U0126-treated HSECs demonstrated a decreased expression of NRF1 and NRF2 mRNA and protein on Day-7. TFAM protein and mRNA levels continued to rise on Days-7 and -14 in the control ALI culture. Although similar TFAM expression trends were observed in the ALI culture of Si-WDPCP HSECs and U0126-treated HSECs, there was a decrease in the protein level in Si-WDPCP HSECs on Day-14 compared with controls. In the control group, COX4 expression was significantly increased on Day-7. However, the COX4 gene and protein levels were reduced in Si-WDPCP HSECs on Days-7 and -14, and COX4 protein expression was also decreased in U0126-treated HSECs on Day-7 (Figure 4).

### WDPCP Stabilized Mitochondrial Function Through the MAPK/ERK Pathway

**Figure 5:**
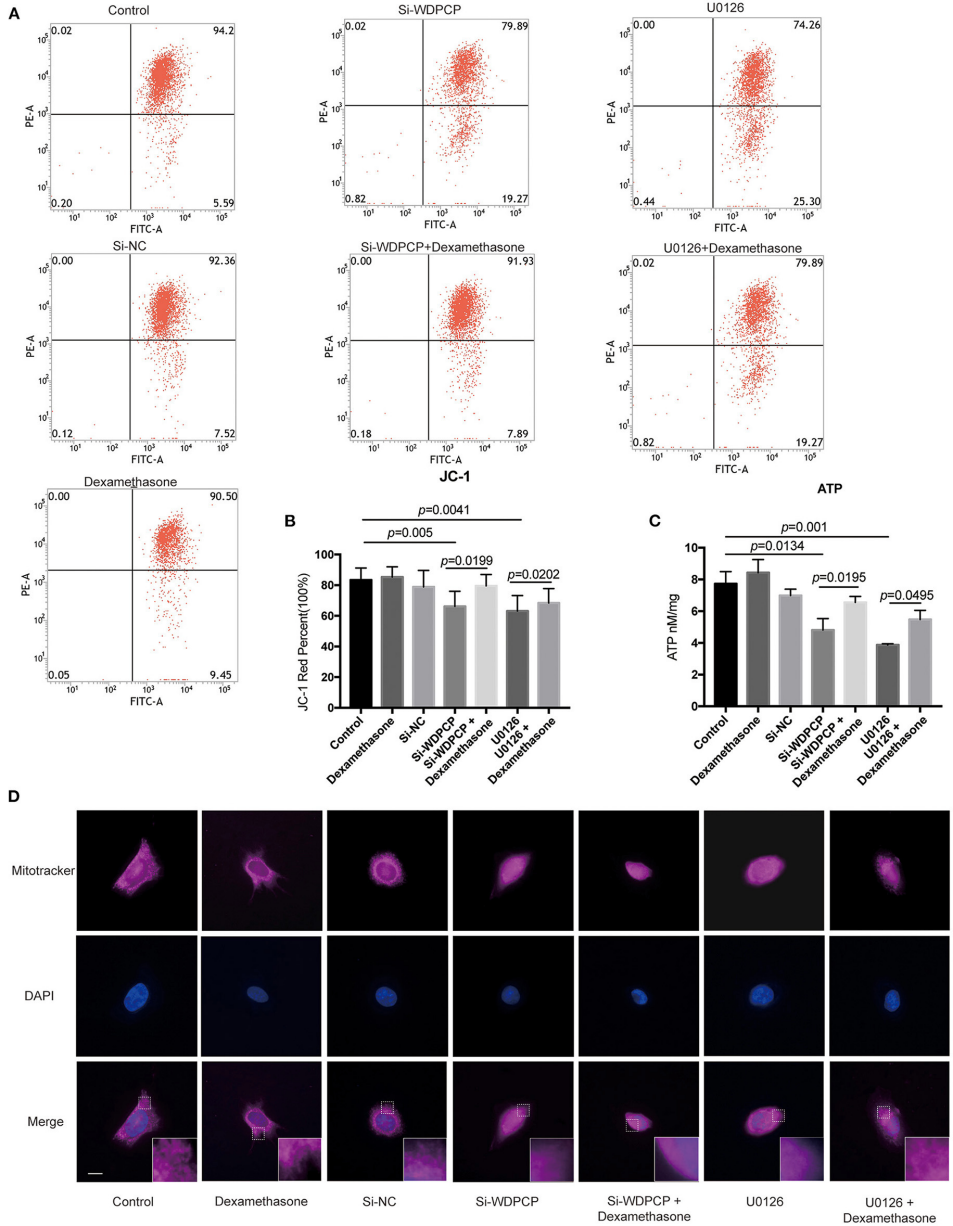
WDPCP impaired mitochondrial function in HSECs by the MAPK/ERK pathway. **(A,B)** The mitochondrial membrane potential was decreased in Si-WDPCP HSECs and U0126-treated HSECs as observed by JC-1 fluorescent dye analysis, and the mitochondrial membrane potential could be partially restored after adding dexamethasone (*n* = 5). **(C)** ATP levels were decreased in Si-WDPCP HSECs and U0126-treated HSECs; after adding dexamethasone, the ATP levels increased (*n* = 5). **(D)** MitoTracker staining showed that rod-shaped mitochondria were decreased in Si-WDPCP HSECs and U0126-treated HSECs, and the mitochondrial morphology cannot be restored by dexamethasone. Scale bar: 8 μm.

In addition to the number of mitochondria, the function of mitochondria is also critical for the beating of cilia (Burkhalter et al., 2019). Flow cytometry analysis revealed a depolarization of the mitochondrial membrane in Si-WDPCP HSECs and U0126-treated HSECs (Figures 5A,B). We also measured mitochondrial function by analyzing ATP production, and we found that ATP levels were reduced in Si-WDPCP HSECs and U0126-treated HSECs (Figure 5C). Interestingly, dexamethasone could repair the damage of mitochondrial function caused by the lack of WDPCP as well as inhibition by U0126 (Figures 5A–C). To monitor mitophagy in the HSECs, mitochondria were visualized with fluorescent MitoTracker. From the MitoTracker staining, rod-shaped mitochondria can be detected in the control and Si-NC group, but not in the Si-WDPCP or U0126 group. Despite the use of dexamethasone, the mitochondrial structure was difficult to recover (Figure 5D).

## Discussion

Patients with CRSwNP were subjected to repeated cycles of inflammation and infection, which resulted in severe cilia loss and increased mucus secretion. In addition to direct ciliary loss, the surviving cilia that experienced inflammatory and/or microbial damage appeared to be dysfunctional. However, the reason behind poor ciliation and cilia dysfunction is still unclear. PCP pathway proteins, such as Intu, Vangl2, and WDPCP were important units that controlled ciliogenesis and cilia function. Studies showed that WDPCP governs ciliogenesis in *Xenopus* embryos (Cui et al., 2013; Park et al., 2015) and human nasal epithelium (Ma et al., 2017). Although short cilia might contribute to cilia beating dysfunction (Bottier et al., 2019), it is still unclear whether WDPCP directly regulated the beating of cilia. To our knowledge, this is the first study of WDPCP on cilia function in human sinonasal epithelium. Our data showed that the low expression of WDPCP in nasal polyps was accompanied with a decreased frequency of cilia beating. Following WDPCP silencing in ALI cultures of HSECs, there was a decrease in CBF. These data suggest that in addition to its role in the ciliogenesis of HSECs, WDPCP is also critical to cilia beating function.

Mitochondria are highly dynamic organelles that are essential for energy production and cell homeostasis. Mitochondrial function modulates cell survival, metabolism, and health status. The impairment of mitochondrial function has been involved in a variety of pathological conditions and diseases. For example, mitochondrial dysfunction could provoke heterotaxy *via* aberrant ciliogenesis and reduced the beating of cilia (Burkhalter et al., 2019). Our finding showed that the expression of mitochondrial biogenesis markers such as NRF1, NRF2, TFAM, and COX4 was decreased in the epithelium of nasal polyp. HSECs that lacked the expression of WDPCP had decreased mtDNA copy number; low expression of NRF1, NRF2, TFAM, and COX4; and poor mitochondrial function and structure. However, the mitochondrial apoptosis markers were not activated. Overall, these findings support the notion that WDPCP-regulated cilia function from its effects on the mitochondria.

**Figure 6:**
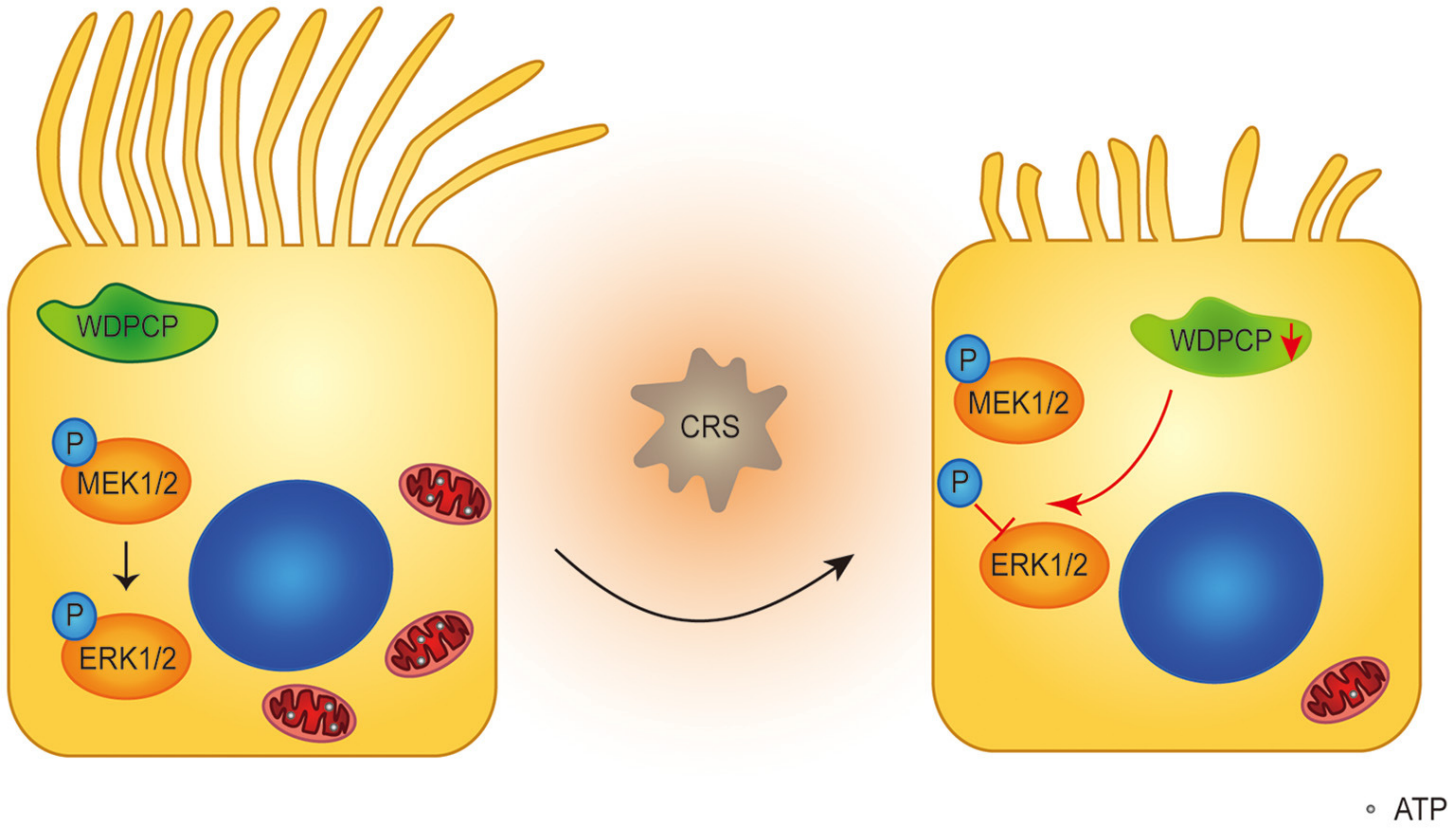
Schematic diagram of WDPCP regulating ciliogenesis and cilia function in nasal epithelium. Our results showed that WDPCP could work through MAPK/ERK pathway to regulate mitochondrial biogenesis and function. In the case of CRSwNP, HSECs low expressed WDPCP, and phosphorylation of ERK1/2 was partially blocked, further affecting the number and function of mitochondria. This mechanism might lead to the poor ciliation and cilia dysfunction of nasal epithelium in CRSwNP.

The MAPK/ERK pathway regulates a series of cellular biological behaviors. Although the importance of MAPK/ERK activity in morphogenesis and metabolism is emerging (Boucherat et al., 2014), the role of the MAPK/ERK pathway in ciliogenesis and the function of human airway epithelium is still unknown. Our results showed that after WDPCP silencing, the expression of p-ERK in HSECs was decreased. Also, when MAPK/ERK activity was inhibited by U0126, HSECs developed fewer and shorter cilia in ALI cultures, a phenotype reminiscent of WDPCP deficiency. Moreover, in the absence of MAPK/ERK activity, the mtDNA copy number and the expression of NRF1, NRF2, and TFAM were decreased in HSECs, and the structure of mitochondria was altered. Collectively, these results demonstrate that WDPCP acts through the MAPK/ERK pathway to regulate ciliogenesis and mitochondrial biogenesis (Figure 6). Consistent with our findings, the role of MAPK/ERK activity in cellular differentiation has already been investigated. For example, MAPK/ERK activation was involved in the differentiation of nephron progenitors by the Wnt and Notch pathways (Ihermann-Hella et al., 2018), in addition to the crucial role of the MAPK/ERK pathway in airway development (Boucherat et al., 2014). Our findings highlight the importance of MAPK/ERK activity in airway ciliogenesis and cilia function.

Glucocorticoids and their receptors could modulate mitochondrial function. Glucocorticoids activated nuclear-encoded genes, which resulted in enhanced mitochondrial biogenesis (Goffart and Wiesner, 2003; Psarra and Sekeris, 2011). Additionally, glucocorticoids could directly regulate mitochondrial function by interpolating into the mitochondrial membrane (Sionov et al., 2006; Lee et al., 2013). Hence, we speculated that glucocorticoids could also repair mitochondrial damage in airway diseases. In order to address this hypothesis, we examined the expression of mitochondrial biogenesis markers and mitochondrial function after treatment with dexamethasone in Si-WDPCP HSECs and U0126-treated HSECs. Interestingly, we found that the decreased NRF1 in HSECs that lacked WDPCP expression could be repaired by dexamethasone. However, dexamethasone could not repair the mitochondrial structure. Taken together, dexamethasone could partially recover the damage on mitochondrial function in HSECs that lacked WDPCP expression or HSECs with an inactivated MAPK/ERK pathway. These findings provide a new insight into the mechanism underlying glucocorticoid treatment of CRSwNP.

### Limitation

To our knowledge, the research of metabolism in the nasal mucosal epithelium is very limited. Our research provides ideas for revealing mucosal cilia dysfunction in patients with CRSwNP. However, there are still some limitations. First, most of our results were based on the study *in vitro*, which might be different from the real world of the nasal mucosa in CRSwNP patients. Secondly, our research explored the effects of glucocorticoids in nasal epithelium, but little on the mitochondrial; more in-depth research is still needed to explain the therapeutic effects of glucocorticoids in CRSwNP. Thirdly, we found that WDPCP-regulated cilia function through its effects on the mitochondria but does not activate the mitochondrial apoptosis; more research is needed to explore the deep mechanism.

## Conclusions

In summary, WDPCP regulates the mitochondrial biogenesis and mitochondrial function through the MAPK/ERK pathway to affect cilia-beating function. Dexamethasone could partially repair the mitochondrial damage. Further investigation of PCP proteins may offer a deeper understanding of the pathophysiology and treatment of CRSwNP.

## Data Availability Statement

The original contributions presented in the study are included in the article/Supplementary Material, further inquiries can be directed to the corresponding author/s.

## Ethics Statement

The studies involving human participants were reviewed and approved by Ethics Committee of Sun Yat-sen Memorial hospital, Sun Yat-sen University. The patients/participants provided their written informed consent to participate in this study.

## Author Contributions

YM, HZh, HZo, and YZ designed the experiments. YM and PT did experiments on Western blot, real-time PCR, and cell culture. HZh performed tests on cilia beating frequency and immunostaining. FW and QZ were responsible for mitochondrial related experiments. YM, HZh, and PT analyzed the data and wrote the manuscript. XL, HD, QC, and HZo recruited patients and collected specimen. All authors contributed to the article and approved the submitted version.

## Conflict of Interest

The authors declare that the research was conducted in the absence of any commercial or financial relationships that could be construed as a potential conflict of interest.
