# Supplementary material for: WDPCP Modulates Cilia Beating Through the MAPK/ERK Pathway in Chronic Rhinosinusitis With Nasal Polyps
Source: Front Cell Dev Biol. 2021 Feb 1;8:630340. doi: 10.3389/fcell.2020.630340 (PMC7882705; doi:10.3389/fcell.2020.630340)
Supplement: Supplementary file 1 [file Table_1.DOCX]

**Table S1** The primer sequences (5’-3’) for each gene

| Gene | Primer sequences | |
| --- | --- | --- |
| *WDPCP* | Forward primer | ATTGCGGATAGAGACATTGGGA |
|  | Reverse primer | TGTTTTTGAGCGTCCAAGGATAA |
| NRF-1 | Forward primer | AGTGCTTAGCCCTTGATGAAGA |
|  | Reverse primer | GCTCTGAAGTGACCTCTGGTAT |
| NRF-2 | Forward primer | GGCGTTAGAAAGCATCCTTCC |
|  | Reverse primer | GCAGAGGGCACACTCAAAGT |
| TFAM | Forward primer | ATGGCGTTTCTCCGAAGCAT |
|  | Reverse primer | TCCGCCCTATAAGCATCTTGA |
| COX4 | Forward primer | CCTTCTGCACAGAACTCAACGC |
|  | Reverse primer | AGGTCTCATTGAACTGGAGCCG |

**
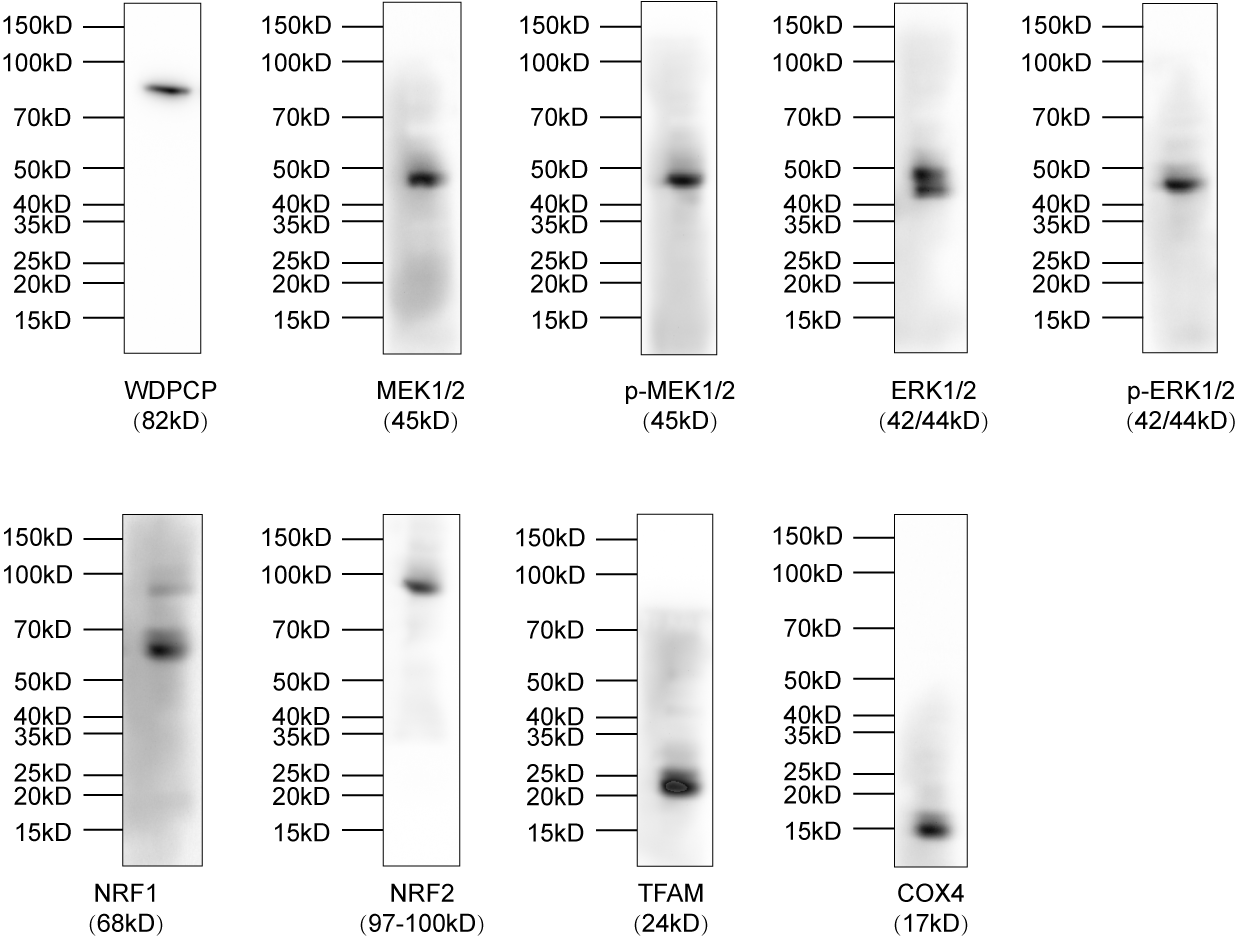
**

**Figure S1.** Antibody specificity test by Western blot. The bands of Western bolt showed that all the antibodies have good specificity.

**Figure S2.** Transfection efficiency of Si-RNA. Under the conditions of Si-RNA concentration of 200 nM and lipo 3000 concentration of 20 μg/mL, the survival rate of primary human nasal mucosal epithelial cells is good and the transfection efficiency can reach 77.74±1.82%.

**Video Legends**

Video 1. Cilia beating video of control mucosa.

Video 2. Cilia beating video of nasal polyp.

Video 3. Cilia beating video of control HSECs in ALI

Video 4. Cilia beating video of Si-WDPCP HSECs in ALI

Video 5. Cilia beating video of U0126 treated HSECs in ALI
